# Supplementary material for: Patterns of Sequelae in Women with a History of Localized Breast Cancer: Results from the French VICAN Survey
Source: Cancers (Basel). 2021 Mar 8;13(5):1161. doi: 10.3390/cancers13051161 (PMC7962808; doi:10.3390/cancers13051161)
Supplement: Supplementary file 1 [file cancers-13-01161-s001.pdf]

Supplementary data

Table S1: Description of the key words and expressions used by the BC women from the VICAN study (n=654) to recode the variables into 21 sequelae.

Sequelae	Keywords/expressions
Functioning	
Arm	Loss of hand sensitivity<br>Numbness in the hands<br>Arm disability<br>Difficulty in raising the arm<br>Difficulty carrying things<br>Loss of strength in the arm<br>Stiffness in the arm<br>Hand swelling<br>Oedema<br>Reduced amplitudes
Other	Difficulty to move<br>Back problem<br>Muscular pain<br>Breast sensitivity<br>Impossible to bend down<br>Difficulty in making an effort<br>Shivers all the time<br>Head spinning<br>Recovery time<br>Disability<br>Overall loss of strength/legs<br>I can no longer carry any weight<br>Difficult to walk<br>Without medication I can no longer walk<br>Loss of motricity<br>Heavy members<br>Loss of energy<br>I can no longer do the same physical activities as before
Pain	Pain<br>Excruciating tingling<br>Stab<br>Suffering<br>Sensitivity<br>All the time bad
Esthetic	Scar<br>Difference in breast volume/asymmetry<br>Hair that doesn't grow back like it used to<br>Breast deformity<br>I can't look at myself in the mirror<br>Skin colour problem (discolouration or darker skin)<br>Loss of breast(s)<br>Loss of nails<br>Hair that is different<br>Depigmentation<br>Eyebrows that no longer grow back<br>Mutilation
Psychological	Psychologically it's difficult<br>Too much stress

	Fear of relapse<br>Fear of dying<br>Worried about the future<br>Suicidal thoughts<br>Low morale<br>Declining confidence<br>I can't look at myself in the mirror<br>Difficulty in accepting the situation<br>Anxiety<br>Psychological sequelae<br>Difficult to live with<br>They don't spare me<br>Phantom breast<br>We think about illness every day<br>I feel abandoned<br>I often cry<br>Different life<br>Depression<br>I see things negatively<br>I can't forget what happened<br>Upheaval in life<br>No longer be like the others<br>Mutilation<br>Loneliness
Gynecological	Hot flush<br>Vaginal dryness<br>Burning sensations<br>Excessive perspiration<br>Gynecological problems
Fatigue	Fatigue<br>I need to rest often<br>I take naps<br>Lack of sleep
Cognitive	Concentration problem<br>Memory problem<br>Neurological trouble<br>I'm losing my mind
Weight	Put on weight<br>I have to pay attention to my figure
Urinary	Urinary infections
Skin	Skin reaction<br>Skin thickening<br>Skin burn<br>Dry skin
Vision	Vision problem<br>Loss of eyesight
Digestive	Digestion trouble<br>Bowel problems
Respiratory	Shortness of breath<br>Loss of breath
Sleep	Insomnia<br>I can no longer sleep<br>Fractioned sleep
Sexual	Difficult married life (sexually speaking)

	Pain during sexual intercourse<br>Loss of libido<br>Difficult to have sex with one less breast<br>Can no longer have children<br>Sexual problem
Cardiac	Cardiac complications
Hearing	Inner ear problem<br>I can no longer hear very well
Financial	I spend a lot of money on my care<br>I can no longer work
Dental	
Other	Sense of smell<br>Taste problems<br>Burn in the mouth<br>Gustative trouble<br>Thyroid disturbance
